# Ovarian Carcinosarcoma With Neuroendocrine Differentiation: A Case Report

**DOI:** 10.7759/cureus.115904

**Published:** 2026-09-07

**Authors:** Aron Bhachawat, Heenal Marfatia, Janira Navarro Sanchez, Suneetha Chintalapati, Locke Uppendahl

**Affiliations:** 1 Radiology, Obstetrics and Gynecology, Kansas City University of Medicine and Biosciences, San Antonio, USA; 2 Family Medicine, Kansas City University, Kansas City, USA; 3 Breast and Gyn Pathology, Ascension Via Christi, Wichita, USA; 4 Genitourinary Pathology, Ascension Via Christi, Wichita, USA; 5 Gynecologic Oncology, Ascension Via Christi, Wichita, USA

**Keywords:** figo stage iiic, metastatic, neuroendocrine differentiation, ovarian carcinosarcoma, peritoneal spread, sarcomatous

## Abstract

We report a case of advanced-stage ovarian carcinosarcoma (OCS) with extensive extra-ovarian involvement and widespread high-grade serous carcinoma involving the omentum, bladder, ileal mesentery, and small bowel serosa in a female patient in her mid-40s. This case highlights a rare arrangement of histologic features and disease differentiation. The metastatic sites demonstrated predominantly epithelial high-grade serous carcinoma, while the sarcomatous component was identified in the adnexal tumor and left pelvic sidewall. The presence of neuroendocrine differentiation, however, represents a rare and infrequently reported histopathologic feature. The case demonstrates the importance of comprehensive histopathologic and immunohistochemical evaluation in accurately characterizing complex gynecologic malignancies.

## Introduction

Ovarian carcinosarcomas (OCSs), also known as mixed malignant Müllerian tumors, are a rare and highly aggressive subtype of ovarian cancer, composed of both malignant epithelial and mesenchymal components within the same tumor. These tumors account for less than 5% of pathologic ovarian carcinomas, often presenting atypically, at an advanced stage of diagnosis, with poor prognosis, and rapid recurrence even after initial treatment [1-3]. Histologically, OCS demonstrates a biphasic architecture consisting of malignant epithelial (carcinomatous) populations, and malignant mesenchymal (sarcomatous) populations [4]. Clinically, these tumors demonstrate highly aggressive behavior. Despite their aggressive clinical behavior, most current studies have focused on patient outcome and survival and have not detailed the histopathological characteristics and spatial patterns of tumor spread [3]. We report a case of OCS with neuroendocrine differentiation, a rare histopathologic feature infrequently described in literature [5]. This tumor demonstrated histologic heterogeneity, with neuroendocrine differentiation within the adnexal tumor and extensive peritoneal involvement consisting predominantly of epithelial metastatic deposits. This combination of differentiation and metastatic distribution is diagnostically noteworthy, as it highlights the importance of comprehensive histopathologic sampling and evaluation in accurately characterizing the components and patterns of spread of OCS. 

## Case presentation

A female patient in her mid-40s with a history of uterine fibroids and prior cholecystectomy, presented with two days of abdominal pain, distention, and decreased appetite. Physical examination revealed abdominal tenderness and distention. Laboratory studies showed anemia and elevated lactic acid levels.

CT of the abdomen and pelvis revealed a fibroid uterus and a large mixed solid and cystic mass occupying the left abdomen, with additional mesenteric implants concerning for metastatic disease. A right pleural effusion was also present. CT-guided biopsy of the abdominal mass was performed. Biopsy of the abdominal mass revealed a poorly differentiated neoplasm. Histologic examination demonstrated a solid, discohesive proliferation of malignant cells with a high nuclear-to-cytoplasmic (N:C) ratio and frequent mitotic figures (Figure 1). Immunohistochemical staining demonstrated diffuse cytoplasmic positivity for synaptophysin, supporting neuroendocrine differentiation (Figure 2). The neoplastic cells were positive for cytokeratin AE1/AE3, PAX8, and synaptophysin, with focal positivity for GATA-3. They were negative for CDX2, cytokeratin 7, cytokeratin 20, p16, TTF-1, ER, chromogranin, and inhibin. Staining for p53 showed an equivocal pattern. The differential diagnosis included renal/genitourinary and ovarian origins, among others.

**Figure 1:**
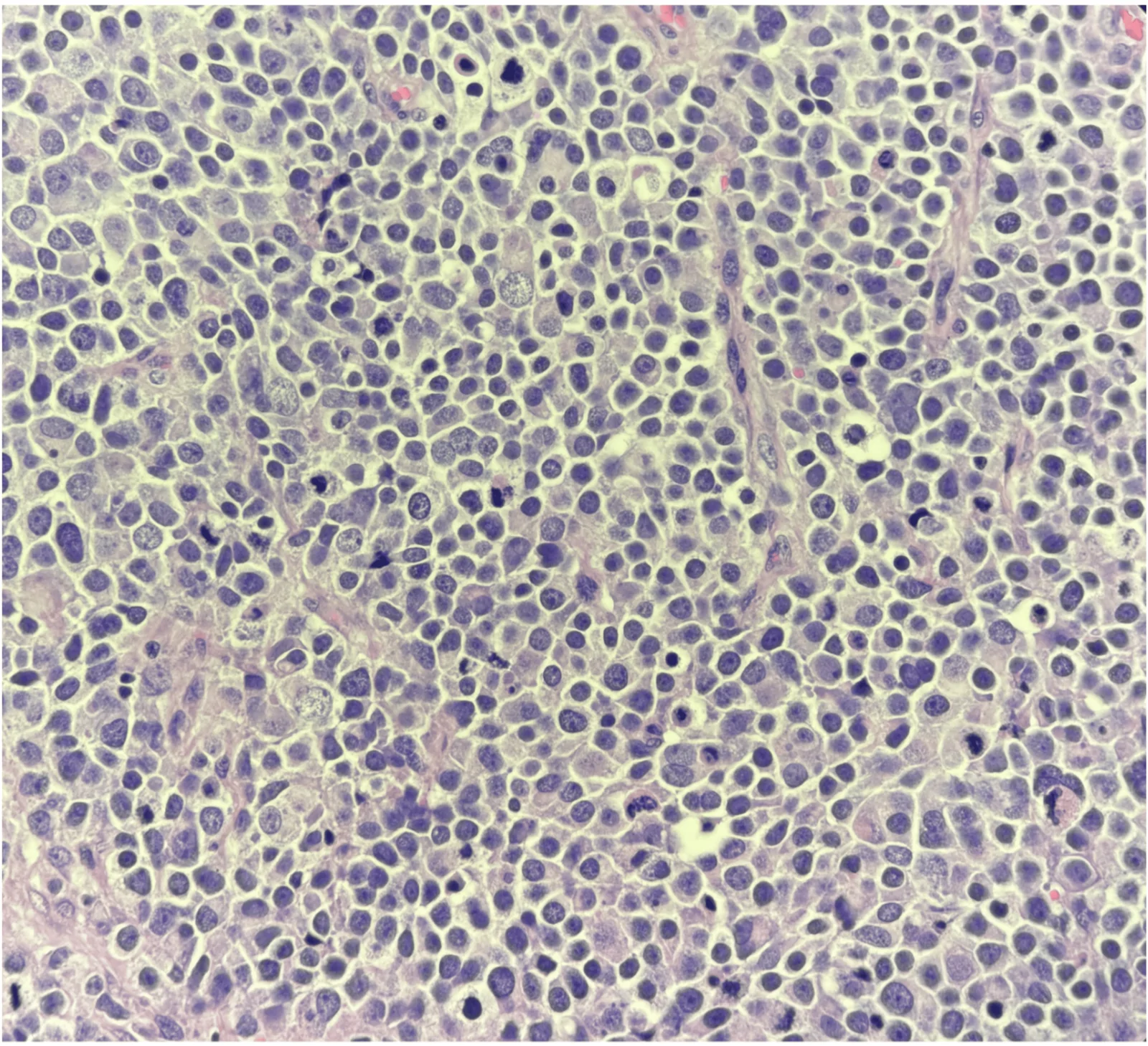
Epithelial component of carcinosarcoma (poorly differentiated carcinoma with neuroendocrine features) showing a solid, discohesive proliferation of cells with high N:C ratio and frequent mitotic figures (H&E stain, 20X magnification) N:C: Nuclear-to-cytoplasmic; H&E: Hematoxylin and eosin

**Figure 2:**
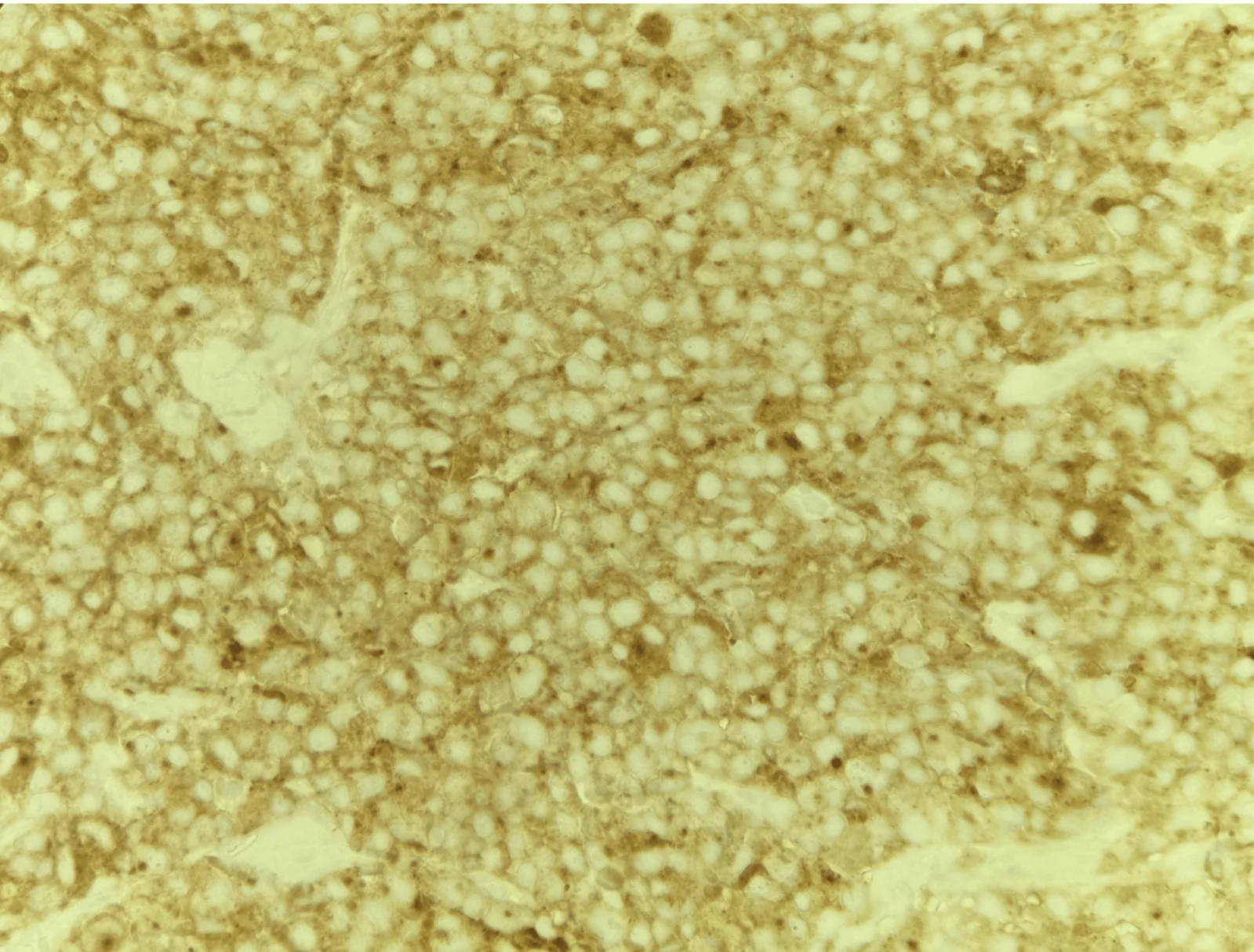
The cells shown are positive for synaptophysin (cytoplasmic staining) (synaptophysin IHC, 20X magnification) IHC: Immunohistochemistry

The patient underwent hysterectomy with bilateral salpingo-oophorectomy, omentectomy, small bowel resection, and resection of pelvic and bladder masses. Pathologic evaluation revealed carcinosarcoma (high-grade serous carcinoma: 90% and undifferentiated sarcoma: 10%) involving left adnexa and right ovary. Histologic examination demonstrated the biphasic morphology characteristic of OCS, with distinct epithelial and sarcomatous components (Figure 3). The epithelial component showed features of high-grade serous carcinoma with papillary architecture (Figure 4). 

**Figure 3:**
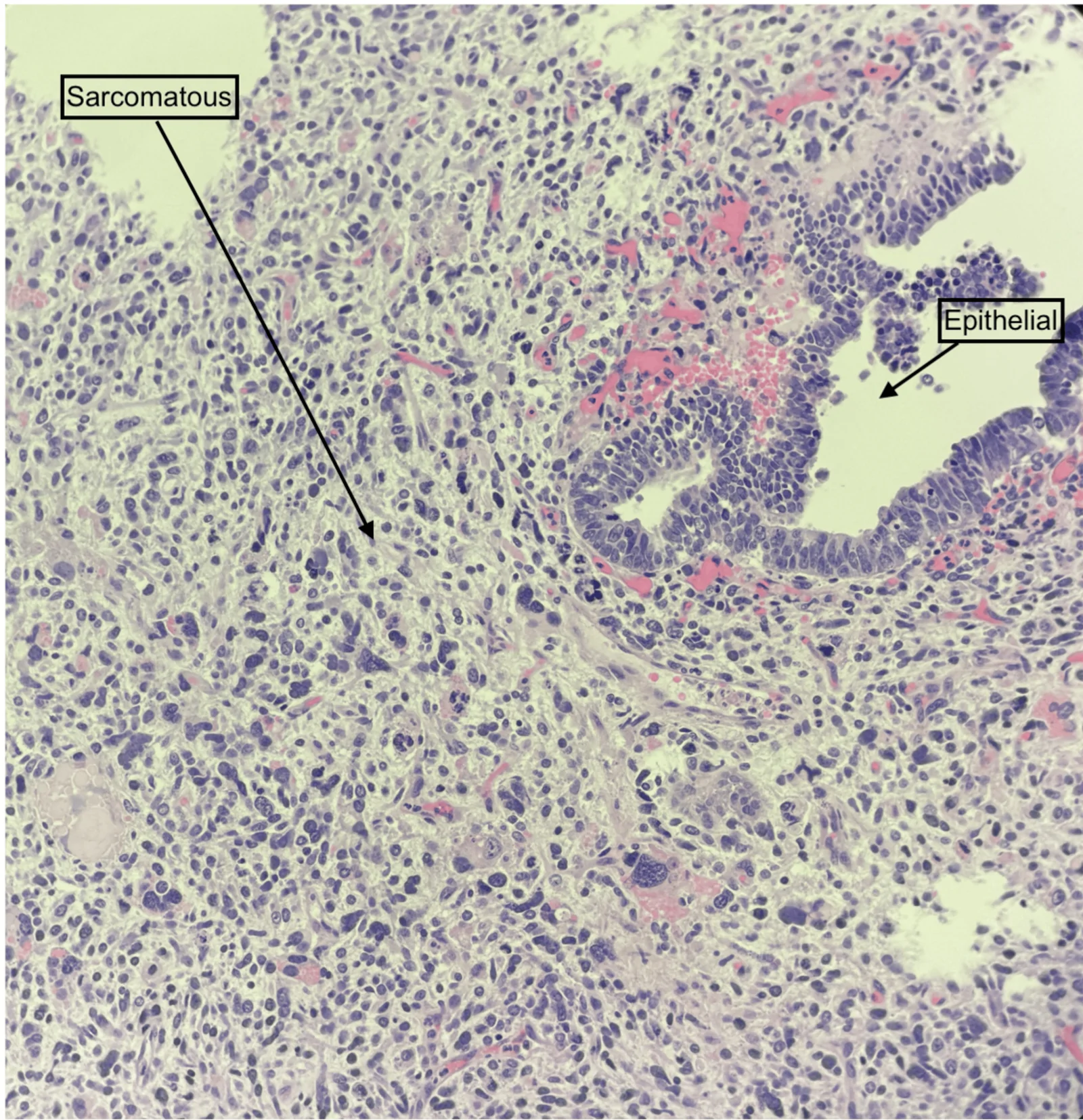
Carcinosarcoma with high-grade serous carcinoma (epithelial component, right) and sarcomatous component (left) (H&E) stain, 10X magnification) H&E: Hematoxylin and eosin

**Figure 4:**
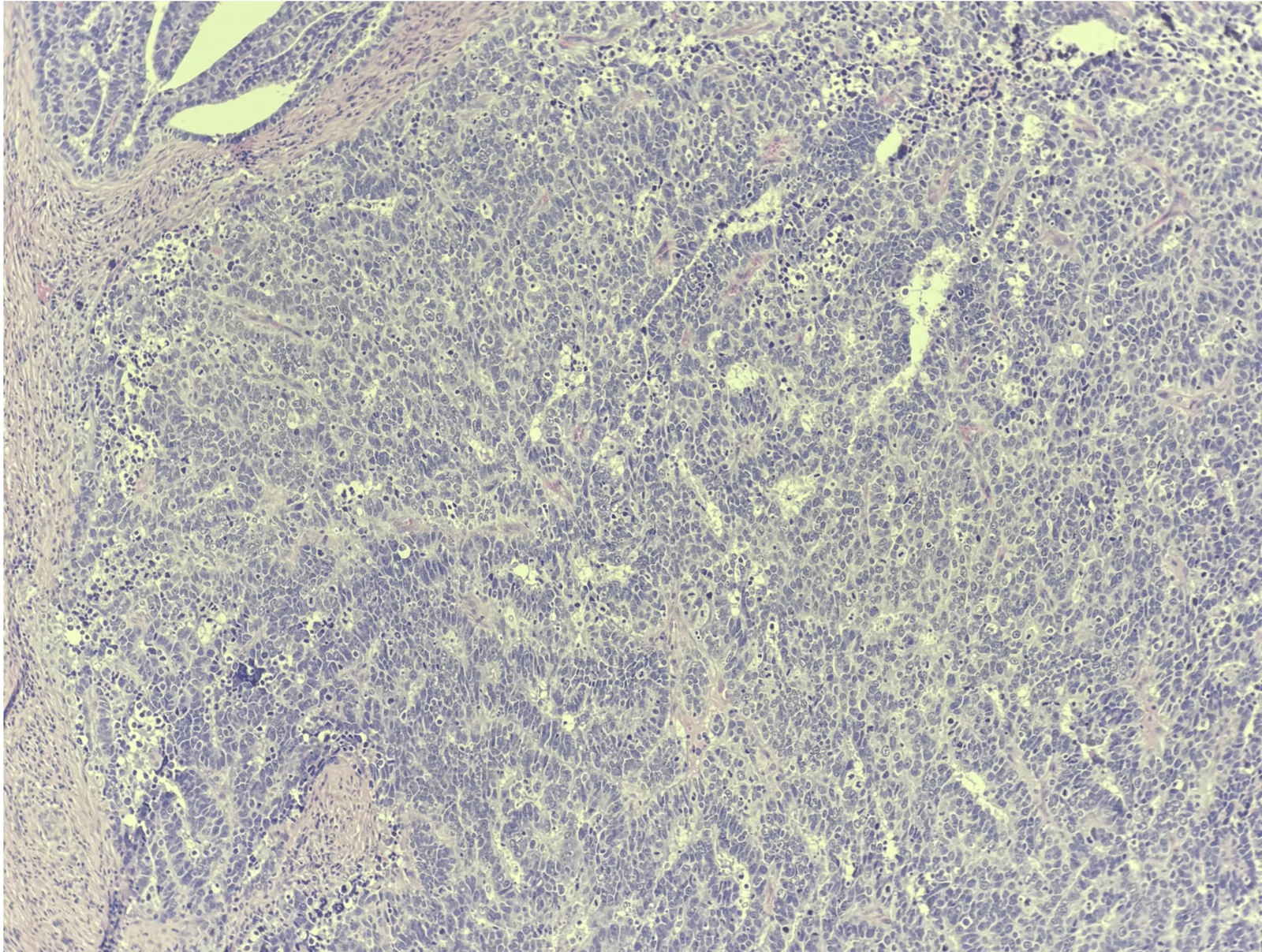
Epithelial component of carcinosarcoma (high-grade serous carcinoma) represented by multiple papillary structures lined by atypical epithelial cells (H&E stain, 10X magnification) H&E: Hematoxylin and eosin

The omentum, bladder, ileal mesentery and small bowel showed involvement by the epithelial component of carcinosarcoma (high grade serous carcinoma). The left pelvic sidewall was involved by carcinosarcoma (predominantly undifferentiated sarcomatous component with separate areas of poorly differentiated carcinoma with neuroendocrine features). 

Immunohistochemical staining showed tumor cell positivity for pankeratin, CK7, PAX8, WT1, CD56, and diffusely strong positive (aberrant) p53 staining. The sarcomatous component demonstrated reduced pankeratin expression associated with areas of poorly differentiated carcinoma with neuroendocrine features showing synaptophysin positivity. One regional lymph node contained isolated tumor cells, and lymphovascular invasion was present. The disease was staged as FIGO stage IIIC (pT3cN0[i+]). Postoperatively, the patient developed ileus and persistent ascites. Follow-up imaging raised concern for possible hepatic metastasis.

## Discussion

OCS is an uncommon but highly aggressive malignancy characterized by rapid disease progression and poor survival outcomes [1,3]. This tumor is a biphasic malignancy composed of high grade carcinomatous and sarcomatous elements, typically presenting at an advanced stage at the time of diagnosis. 80% of the cases at diagnosis have presented with extra-ovarian intra-abdominal spread [3,4,6]. The present case exemplifies this aggressive clinical behavior, with FIGO stage IIIC disease and extensive peritoneal involvement of the omentum, bladder, ileal mesentery, and small bowel serosa identified at the time of surgical exploration.

The pathologic evaluation of this case revealed anadnexal tumor demonstrating the characteristic biphasic architecture of OCS, composed of high-grade serous carcinoma (90%) and undifferentiated sarcoma (10%). Immunohistochemical staining was crucial to delineate these two components. The carcinomatous components demonstrated positivity for pankeratin, CK7, PAX8, WT1, and aberrant p53. Diffusely strong aberrant p53 staining was present, a hallmark of TP53-driven high grade serous carcinoma. This finding supports a shared clonal origin between the epithelial and mesenchymal components [6-8]. In contrast, the sarcomatous component exhibited reduced pankeratin expression, reflecting its mesenchymal differentiation and confirming the biphasic nature of the tumor. A notable finding in this case was the presence of poorly differentiated carcinoma with neuroendocrine features associated with the sarcomatous component in the left pelvic wall resection. This was evidenced by synaptophysin positivity and CD56 expression. Neuroendocrine differentiation in OCS is rare and has only been documented in isolated reports in literature [7]. 

OCSs with neuroendocrine differentiation in the epithelial component are exceedingly rare. A literature review identified only two prior case reports, one from the United States and one from Korea. In these reports, neuroendocrine differentiation was characterized by synaptophysin and chromogranin positivity within the epithelial component, with some exhibiting a predominantly neuroendocrine phenotype. Together, these findings highlight the heterogeneity of epithelial differentiation in OCS [9]. Similar tumors with neuroendocrine differentiation have been described in carcinosarcomas arising at other sites, including the stomach, esophagus, mesentery, and uterine cervix [10,11]. 

The spatial distribution of the histologic components in this case was consistent with previously described patterns of metastatic spread in OCS. Metastatic involvement of the omentum, bladder, ileal mesentery, and small bowel serosa consisted of high-grade serous carcinoma, whereas the left pelvic sidewall demonstrated predominantly undifferentiated sarcoma with separate areas of poorly differentiated carcinoma with neuroendocrine features. This pattern reinforces prior studies that have demonstrated that the epithelial component preferentially drives metastatic dissemination in carcinosarcoma, while sarcomatous differentiation may occur localized [12]. Most existing studies have focused on patient outcomes and survival rather than on detailed characterization of the pathologic features and spatial patterns of tumor spread [6]. 

This case has limitations that are inherent to a single case report. This includes the absence of molecular sequencing to confirm clonal relationships between the carcinomatous and sarcomatous components. In addition, the rare presentation of neuroendocrine differentiation in OCS limits the ability to draw broad conclusions regarding prognostic or biologic significance. Additional studies that incorporate detailed histopathologic and immunohistochemical characterizations of OCS, specifically in cases with unusual features such as neuroendocrine differentiation, are needed to help better understand the pathobiology and clinical implications of this rare malignancy. 

Furthermore, the patient in our case had an updated clinical course. Sheto complete adjuvant chemotherapy and had a CT image that demonstrated no evidence of disease. Unfortunately, three weeks later the patient recurred with an 8 cm tumor and multiple abdominopelvic masses. 

## Conclusions

We describe a rare case of advanced OCS (FIGO stage IIIC) with extensive widespread peritoneal involvement and a rare histopathologic profile. This unusual arrangement highlights the importance of detailed pathologic evaluation and contributes to the limited literature on complex gynecologic malignancies with neuroendocrine differentiation in OCS. Comprehensive immunohistochemical evaluation, including pankeratin, CK7, PAX8, WT1, p53, synaptophysin, and CD56, was essential to accurately delineate the biphasic tumor architecture and identify the rare finding of high grade carcinoma with neuroendocrine differentiation associated with sarcomatous component. The exclusive presence of pure high-grade serous carcinoma at all metastatic sites such as the omentum, bladder, ileal mesentery, and small bowel serosa, with sarcomatous component of carcinosarcoma associated with areas of neuroendocrine differentiation confined to left pelvic side wall, reinforces the importance of the epithelial component in metastatic dissemination and carcinosarcoma histogenesis. The differing distribution of epithelial, sarcomatous, and neuroendocrine features demonstrates the marked histologic heterogeneity that may occur in OCS. 

This case contributes to the limited literature on the histopathologic complexity of OCS and highlights the importance of detailed pathologic and immunohistochemical evaluation in characterizing rare gynecologic malignancies.The clinical and biologic significance of neuroendocrine differentiation in OCS remains uncertain, and additional cases incorporating molecular characterization are needed.
